# Identification of a glycolysis‐related gene signature for survival prediction of ovarian cancer patients

**DOI:** 10.1002/cam4.4317

**Published:** 2021-10-05

**Authors:** Dai Zhang, Yiche Li, Si Yang, Meng Wang, Jia Yao, Yi Zheng, Yujiao Deng, Na Li, Bajin Wei, Ying Wu, Zhen Zhai, Zhijun Dai, Huafeng Kang

**Affiliations:** ^1^ Department of Oncology The Second Affiliated Hospital of Xi'an Jiaotong University Xi'an China; ^2^ Department of Thyroid, Breast and Vascular Surgery Xijing Hospital The Air Force Medical University Xi'an China; ^3^ Department of Tumor Surgery Shaanxi Provincial People's Hospital Xi'an China; ^4^ Department of Breast Surgery The First Affiliated Hospital College of Medicine Zhejiang University Hangzhou China

**Keywords:** bioinformatics, glycolysis, ovarian cancer, prognostic signature

## Abstract

**Background:**

Ovarian cancer (OV) is deemed the most lethal gynecological cancer in women. The aim of this study was to construct an effective gene prognostic model for predicting overall survival (OS) in patients with OV.

**Methods:**

The expression profiles of glycolysis‐related genes (GRGs) and clinical data of patients with OV were extracted from The Cancer Genome Atlas (TCGA) database. Univariate, multivariate, and least absolute shrinkage and selection operator Cox regression analyses were conducted, and a prognostic signature based on GRGs was constructed. The predictive ability of the signature was analyzed using training and test sets.

**Results:**

A gene risk signature based on nine GRGs (*ISG20*, *CITED2*, *PYGB*, *IRS2*, *ANGPTL4*, *TGFBI*, *LHX9*, *PC*, and *DDIT4*) was identified to predict the survival outcome of patients with OV. The signature showed a good prognostic ability for OV, particularly high‐grade OV, in the TCGA dataset, with areas under the curve (AUC) of 0.709 and 0.762 for 3‐ and 5‐year survival, respectively. Similar results were found in the test sets, and the AUCs of 3‐, 5‐year OS were 0.714 and 0.772 in the combined test set. And our signature was an independent prognostic factor. Moreover, a nomogram combining the prediction model and clinical factors was developed.

**Conclusion:**

Our study established a nine‐GRG risk model and nomogram to better predict OS in patients with OV. The risk model represents a promising and independent prognostic predictor for patients with OV. Moreover, our study on GRGs could offer guidance for the elucidation of underlying mechanisms in future studies.

## Graphical abstract

Novel 9‐gene risk signature based on glycolysis‐related genes was developed to predict ovarian cancer survival. A nomogram combining the gene signature and patient characteristics provided superior estimation of overall survival. Our research provided a new prognostic tool and guidelines for future research.

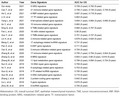

## 1 INTRODUCTION

Among gynecological cancers, ovarian cancer (OV) is considered the most fatal. It is estimated that the respective numbers of new cases and deaths were 22,530 and 13,980 in the United States in 2019.^1^ The main challenges in the development of effective methods for screening and predicting prognosis are attributed to the significant heterogeneity at the clinical, histopathological, and molecular levels of this disease.^2^ Clinical and pathological factors are not sufficient to predict long‐term survival.^3^ An increasing number of opportunities for exploring tumor prognostic markers have emerged, which are attributed to the establishment and development of public biological databases that provide available gene expression data and clinical data of cancers. Many biomarkers, including *EN2* and *HE4* genes, which are associated with the prognosis and survival of OV, have been identified.^4^, ^5^, ^6^, ^7^ With the rapid development of high‐throughput sequencing, a variety of patient genome databases have been constructed to obtain a more systematic understanding of genomic changes. Thousands of prognostic biomarkers have been identified through mining these databases.^8^, ^9^ In addition, studies have found that genetic models constructed using multiple genes have a better prediction performance for cancer prognosis than models based on a single gene.^9^, ^10^ Gene models constructed based on tumor biopsy have practical significance for the guidance of targeted therapy. Currently, several studies have explored the establishment of multigene signatures for assessing the survival risk of patients with OV and predicting clinical outcomes.^8^, ^9^, ^10^, ^11^

Glycolysis occurs in all cells of the body.^12^ A previous study reported that genes involved in glycolysis are ubiquitously overexpressed in 24 cancer classes.^13^ To date, the relationships between glycolysis and the processes of cancer oncogenesis, development, proliferation, and invasion have been the focus of many studies.^14^, ^15^, ^16^ The results from previous studies provide compelling evidence of new glycolysis‐related biomarkers for the prediction of cancer patient survival. Pancreatic cancer patients with a high expression of TCF7L2 have a poorer prognosis than those with low expression levels, and the underlying mechanism is that TCF7L2 positively regulates aerobic glycolysis through the EGLN2/HIF‐1α axis.^17^ Four glycolysis‐related genes (GRGs; *AGRN*, *AKR1A1*, *DDIT4*, and *HMMR*) were identified in a previous study and found to be strongly associated with the clinical outcome of patients with lung adenocarcinoma.^18^ The combination model of nine GRGs has been reported to effectively predict the overall survival (OS) of patients with endometrial cancer.^19^ In addition, a glycolytic gene expression signature score established based on 10 glycolytic genes (*HK2*, *HK3*, *LDHA*, *PKM2*, *GAPDH*, *ENO1*, *LDHB*, *PKLR*, *ALDOB*, and *GALM*) predicts unfavorable clinical outcomes in patients with glioblastoma and is closely associated with the mesenchymal subtype.^14^ However, so far, more researches are needed to explore the predictive value of GRG for the survival of OV patients. A better understanding of the molecular mechanisms of OV can help in the development of more effective targeted therapies that contribute to improved prognosis.

In this study, we aimed to investigate specific GRG markers that are closely associated with the survival of patients with OV using data from The Cancer Genome Atlas (TCGA; https://portal.gdc.cancer.gov/) database and evaluate the prognostic value of these biomarkers for the prediction of survival in patients with OV. An effective 9‐GRG risk predictive model was constructed to predict the survival outcomes in patients with OV. Notably, the GRG risk model enabled identification of patients with poor prognoses in the high‐risk group. The results of multivariate Cox regression analyses implied that our risk model effectively predicted OS in patients with OV, independent of clinical factors.

## 2 MATERIALS AND METHODS

### 2.1 Data collection

We extracted clinical and RNA sequencing data of patients with OV from TCGA (https://portal.gdc.cancer.gov/). The exclusion criteria were as follows: (1) confirmed non‐OV pathological diagnosis and (2) OV patients with incomplete information regarding clinical characteristics (age, tumor stage, histological grade, survival time, and status). Finally, the total clinical information of 583 patients from the TCGA cohort was collected. The patients from TCGA were defined as a training cohort, whereas datasets from the Gene Expression Omnibus database (http://www.ncbi.nlm.nih.gov/geo/) were selected as external validation sets to validate the robustness of the DRG prognostic model. The sets for validation included GSE63885, GSE26193, and GSE30161 datasets, and their expression profiles were all based on the GPL570 platform; these three cohorts contained 101, 107, and 58 OV samples, respectively.^20^, ^21^, ^22^ The GRG sets were obtained from the Molecular Signatures Database (MSigDB, http://www.gsea‐msigdb.org/gsea/msigdb/index.jsp).^23^

### 2.2 Construction and evaluation of the nine‐GRG prediction model

We applied a log2 transformation to standardize the expression of each gene. We used a *p* < 0.05 as the screening criterion and performed univariate Cox analysis, the least absolute shrinkage and selection operator (LASSO) method,^24^, ^25^ and multivariate Cox regression analysis to identify the best gene model using the R package “glmnet.”^25^, ^26^ Based on the Akaike information criterion, the best GRG combination was selected to construct a predictive model.^27^ We calculated the risk score using the following formula: risk score = $\sum_{i=1}^{n}\text{coef}*\text{id}$.^28^ The Kaplan‐Meier survival curve constructed using the R package “survival”^29^, ^30^ demonstrated the OS of the high‐ and low‐risk groups, which were stratified according to median risk score. The time‐dependent receiver operating characteristic (ROC) curve was used to assess the performance of the gene risk model and compare the prediction efficiency with clinical features or single genes using the R package “survivalROC.”^29^ Univariate and multivariate Cox regression analyses were performed to determine the prognostic value of the signature and some clinicopathological features. To estimate the likelihood of survival, a nomogram was constructed using the R package of “rms”^31^ based on the risk score and clinical features that were analyzed after multivariate Cox regression analysis.

### 2.3 Statistical analyses

We compared the distribution of the clinical features, which included age, tumor stage, and histological grade, between the different subgroups by using chi‐square tests. We used the R version 3.6.2 software to conduct the statistical analyses. Statistical analyses were performed using the R packages “survivalROC,” “survival,” “glmnet,” and “rms.”^25^, ^26^, ^29^, ^30^, ^31^ Statistical differences were deemed significant when the *p*‐value was less than 0.05.

## 3 RESULTS

### 3.1 Patient characteristics and collection of GRGs

We downloaded the complete data of 583 patients with OV, including clinical information and the expression profiles of RNA sequencing, from the TCGA database. We manually searched for GRG sets from MSigDB version 6.2 and referenced the relevant literature. Five related gene sets (REACTOME_GLYCOLYSIS, HALLMARK_GLYCOLYSIS, GO_GLYCOLYTIC_PROCESS, KEGG_GLYCOLYSIS_GLUCONEOGENESIS, and BIOCARTA_GLYCOLYSIS_ PATHWAY) were downloaded, and 443 genes were obtained. After excluding duplicate genes, 386 genes were retained for subsequent analysis. The integrated clinical data and list of GRGs are shown in Table 1 and Table S1, respectively.

**TABLE 1:** Clinic pathological characteristics of extracted patients with ovarian cancer

Characteristic	Group	No. of cases (%)
Age (years)	≤65	403 (69.13)
	>65	180 (30.87)
TNM stage	Stage I	33 (5.66)
	Stage II	41 (7.03)
	Stage III	389 (66.7)
	Stage IV	102 (17.50)
	Stage X	18 (3.08)
Histologic grade	G1	11 (1.89)
	G2	101 (17.32)
	G3	456 (78.21)
	G4	1 (0.17)
	GX	14 (2.40)
Vital status	Alive	241 (41.34)
	Dead	342 (58.66)

Abbreviations: GX, unknown histological grade; Stage X, unknown pathological stage.

### 3.2 Construction of the glycolysis‐related risk signature

**FIGURE 1:**
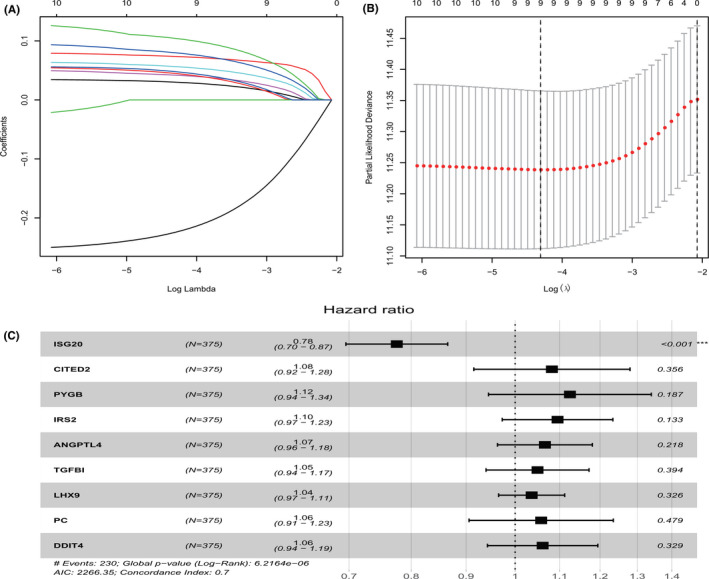
GRGs selection using the LASSO model. (A) Ten‐fold cross‐validation for the coefficients of 11 GRGs in the LASSO model. (B) X‐tile analysis of the nine selected GRGs. (C) Forest plot illustrating the multivariable Cox model results of each gene in nine‐GRG risk signature. GRGs, glycolysis‐related genes; LASSO, the least absolute shrinkage and selection operator cox; OV: ovarian cancer

**FIGURE 2:**
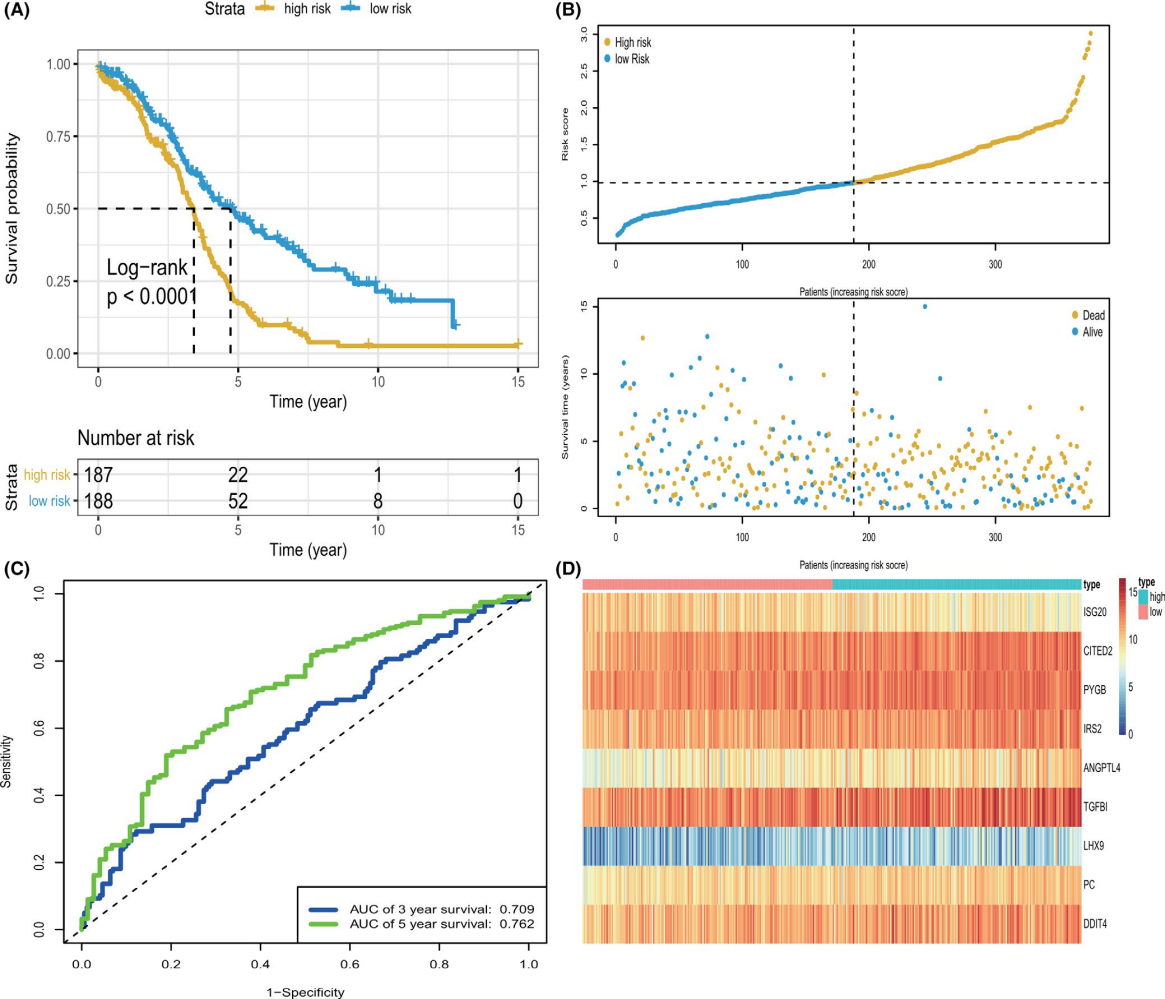
KM survival analysis, risk score assessment by the GRG risk signature and time‐dependent ROC curve in the training set. (A) KM survival analysis of high‐ and low‐ risk samples in the TCGA dataset. (B) Relationship between the survival status/risk score rank and survival time (years)/risk score rank. (C) Time‐dependent ROC curve for OS of the TCGA dataset. The AUC was assessed at 3and 5y. (D) Nine GRGs expression patterns for patients in high‐ and low‐risk groups by the nine‐GRG signature. GRGs, glycolysis‐related genes; OS, overall survival

Among the 386 GRGs, only 201 genes overlapped with those from the OV TCGA RNA sequencing data. To further examine the prognostic value of these genes, we first performed univariate Cox regression analysis, and only 11 genes were obtained by preliminary screening using the criterion of adjusted *p* < 0.05. LASSO analysis was conducted to minimize overfitting (Figure 1A,B). Finally, nine genes (*ISG20*, *CITED2*, *PYGB*, *IRS2*, *LHX9*, *PC*, *ANGPTL4*, *TGFBI*, and *DDIT4*) were screened after multivariate Cox regression analysis (Figure 1C; Table 2). Then, the best predictive signature based on nine GRGs was constructed, and the formula to assess the survival risk of every patient was calculated as follows: risk score = (−0.25414) × *ISG20* expression level + 0.07897 × *CITED2* expression level + 0.11769 × *PYGB* expression level + 0.09112 × *IRS2* expression level + 0.06399 × *ANGPTL4* expression level + 0.04811 × *TGFBI* expression level + 0.03555 × *LHX9* expression level + 0.05593 × *PC* expression level + 0.05907 × *DDIT4* expression level. The gene model was used to calculate each patient's risk score in the training set based on the expression level of the nine GRGs, and all the patients in the training set were classified into either high‐risk or low‐risk group according to their median risk score. The results of Kaplan‐Meier survival analysis suggested that the prognosis of patients with high‐risk scores was worse than that of patients with low‐risk scores (*p* < 0.0001, log‐rank test; Figure 2A). As shown in Figure 2B, we ranked the risk scores of patients in the training set and analyzed their distribution. The survival status of patients with OV in the training set was marked on a dot plot (Figure 2B). The areas under the curve (AUC) for the 3‐ and 5‐year OS were 0.709 and 0.762, respectively (Figure 2C). The heatmap revealed the differences in expression patterns of the nine GRGs between the two prognostic patient groups (Figure 2D).

**TABLE 2:** Coefficients and multivariable Cox model results of each gene in 9‐GRG risk signature

Gene	Ensemble ID	Coefficient	HR	*p* value
ISG20	ENSG00000172183	−0.25414	0.78	5.22E−06
CITED2	ENSG00000164442	0.078975	1.08	0.356061
PYGB	ENSG00000100994	0.117691	1.12	0.186993
IRS2	ENSG00000185950	0.091117	1.10	0.133015
ANGPTL4	ENSG00000167772	0.063993	1.07	0.218495
TGFBI	ENSG00000120708	0.048112	1.05	0.393959
LHX9	ENSG00000143355	0.035546	1.04	0.326248
PC	ENSG00000173599	0.055931	1.05	0.47917
DDIT4	ENSG00000168209	0.059074	1.06	0.328672

Abbreviation: HR, hazard ratio.

### 3.3 Evaluation of the predictive capability of the nine‐GRG risk signature

**FIGURE 3:**
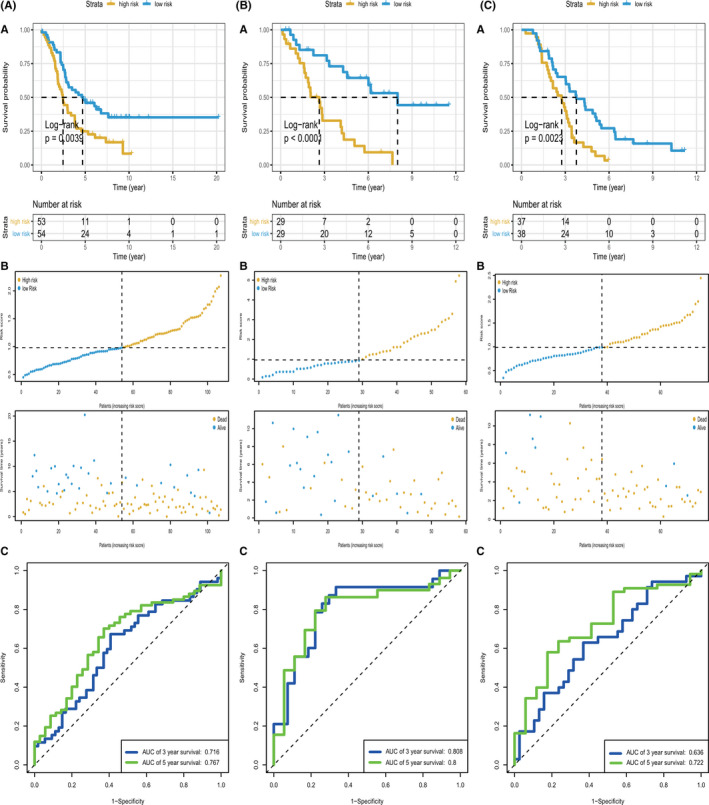
KM survival analysis, risk score assessment by the GRG‐related gene signature and time‐dependent ROC curves in the GEO validation datasets. (A) GSE26193, (B) GSE30161, (C) GSE63885. (a) KM survival analysis of high‐ and low‐risk samples. (b) Relationship between the survival status/risk score rank and survival time (years)/risk score rank. (c) ROC curve for overall survival of the validation datasets. The AUC was assessed at 3 and 5 years

**FIGURE 4:**
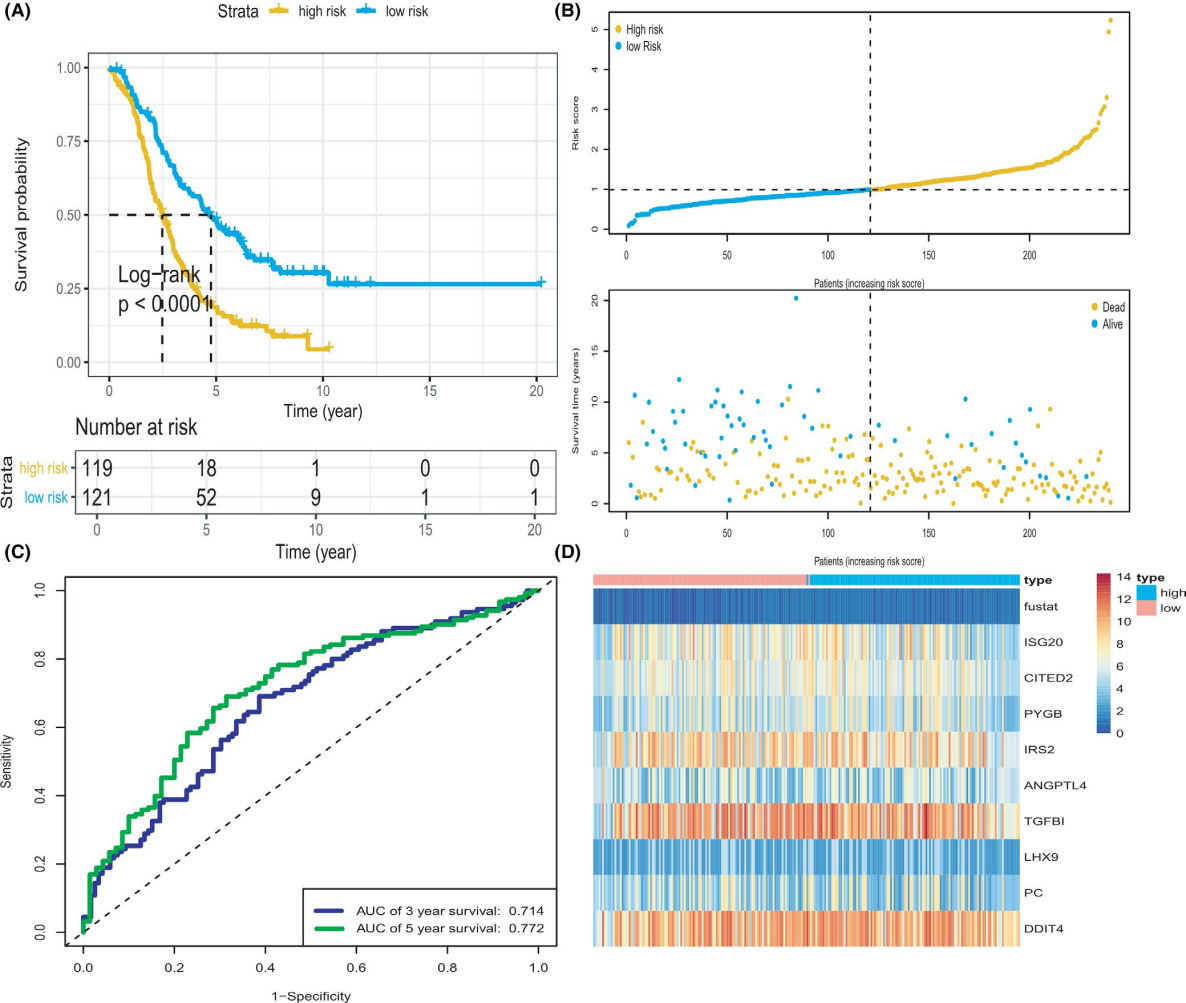
KM survival analysis, risk score assessment by the GRG risk signature and time‐dependent ROC curve in the combined set. (A) KM survival analysis of high‐ and low‐risk samples in the combined set. (B) Relationship between the survival status/risk score rank and survival time (years)/risk score rank. (C) Time‐dependent ROC curve for OS of the combined set. The AUC was assessed at 3 and 5 years. (D) Nine GRGs expression patterns for patients in high‐ and low‐risk groups by the nine‐GRG signature

After constructing the GRG predictive model, we selected three datasets to verify the prediction performance. The sets for validation included GSE63885, GSE26193, and GSE30161 datasets of 101, 107, and 58 patients with OV, respectively. The demographic and clinical characteristics of patients with OV in the validation datasets are presented in Table S2. The AUCs of 3‐ and 5‐year OS were, respectively, 0.716 and 0.767 in the GSE26193 dataset (Figure 3Ac); 0.808 and 0.800 in the GSE30161 dataset (Figure 3Bc); and 0.636 and 0.722 in the GSE63885 dataset (Figure 3Cc). Survival analysis revealed that our risk signature performed well in the validation sets. The survival differences between the high‐risk and low‐risk groups were statistically significant in the GSE63885 cohort (*p* = 0.0039). Similarly, in the GSE26193 (*p* < 0.0001) and GSE30161 (*p* = 0.0023) cohorts, the OS for low‐risk patients was higher than that for high‐risk patients. The distribution of risk scores and survival statuses of the patients with OV in the three sets are shown in Figure 3Ab, 3Bb, and 3Cb. In order to get the most optimal estimate of the AUC‐value, we have merged the three validation sets and the survival analysis revealed that our risk signature performed well in the combined validation set (*p* < 0.0001, Figure 4A), and Figure 4B showed the survival status of patients with OV in the combined set. The AUCs of 3‐ and 5‐year OS were 0.714 and 0.772 (Figure 4C). The heatmap revealed the differences in expression patterns of the nine GRGs between the two prognostic patient groups (Figure 4D).

### 3.4 Risk score generated from the nine‐GRG signature as an independent prognostic indicator

**FIGURE 5:**
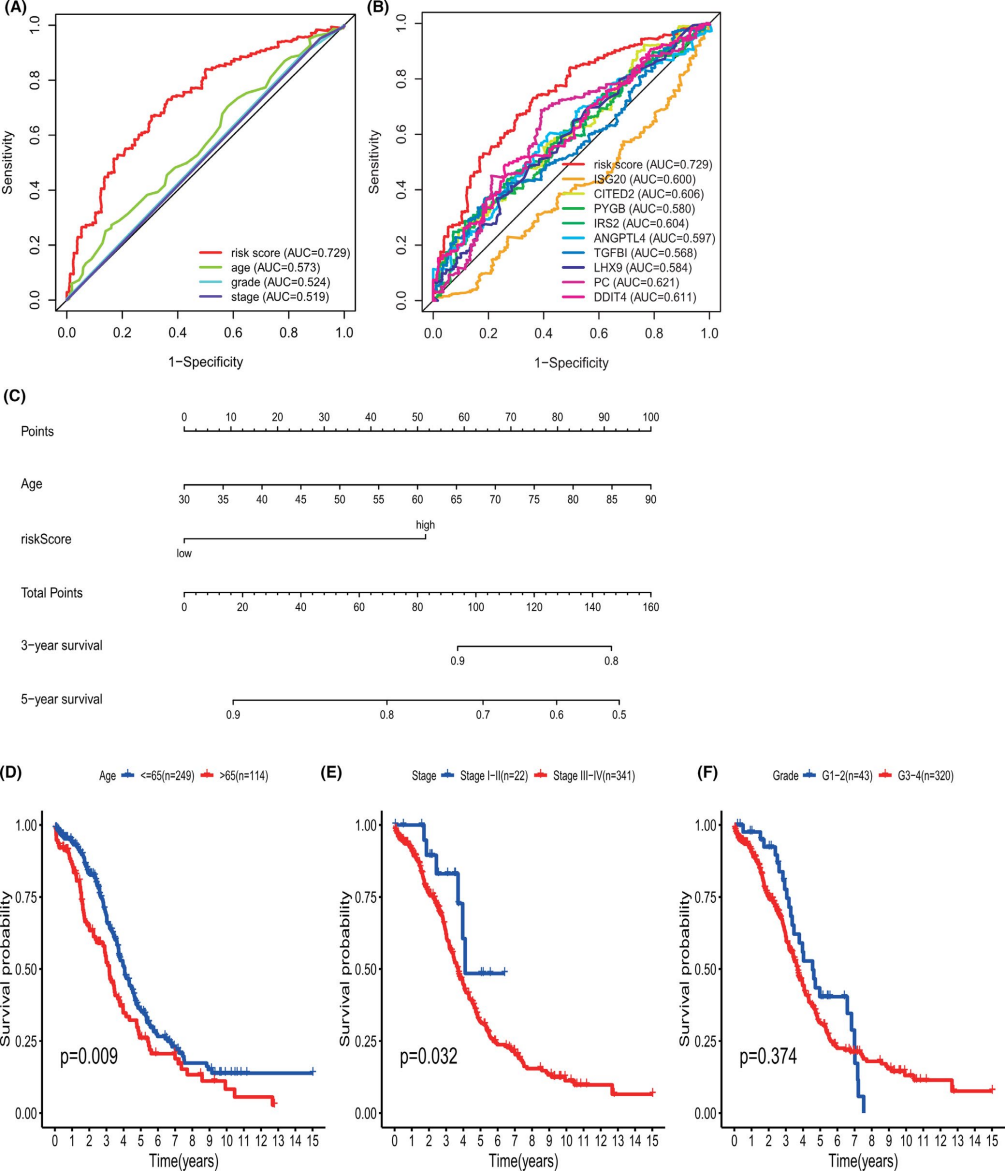
ROC curve with respect to clinical features and risk model, nomogram and Kaplan–Meier survival analysis for OV patients with clinical features: (A) time‐dependent ROC curve with respect to single clinical features and risk model. (B) ROC curves with respect to nine key DRGs in the TCGA cohort. (C) The nomogram for predicting probabilities of OV patients overall survival. Kaplan–Meier survival analysis for OV patients with different clinical features that can predict patient survival (D, Age, E, Stage, F, Grade). OV, ovarian cancer

The exploration of independent predictive factors was performed through univariate analysis of clinical factors and risk models combined with multivariate regression analysis. Table 3 shows that in addition to age and tumor stage, our GRG risk model could independently predict the OS according to the results of univariate analysis (HR [95% confidence interval (CI)], 2.334 [1.817−2.997]; *p* < 0.001) and multivariate analysis (HR [95% CI], 2.361 [1.830−3.047]; *p* < 0.001), referring to the statistical standard of adjusted *p* < 0.05. Furthermore, the AUC of the nine‐gene signature was higher than that of any single clinicopathological variable (Figure 5A). In addition, the AUCs of the time‐dependent ROC curve for the single genes were 0.600 for ISG20, 0.606 for CITED2, 0.580 for PYGB,0.604 for IRS2, 0.597 for LHX9, 0.568 for PC, 0.584 for ANGPTL4, 0.621 for TGFBI, and 0.611 for DDIT4) (Figure 5B), and the sensitivity and specificity of the 9‐gene signature were greater than those of the other single genes. The findings of the present study suggest that the gene model has an independent and effective predictive ability in the survival prediction of patients with OV. A nomogram was constructed to develop a quantitative method that can predict the OS of patients with OV. The predictors included risk score and age. As shown in Figure 5C, points were assigned for each patient characteristic by drawing a line from the scale for each predictor to the point bar at the top of the figure. Then, the points for all predictors were added to determine the total number of points. A patient's predicted probability of having an outcome of interest was determined by drawing a line from the total points bar to the predicted probability bar.

**TABLE 3:** The risk score generated from the nine‐GRG signature as an independent indicator according to Cox proportional hazards regression model

Variable	Univariate analysis		Multivariate analysis	
	HR (95% CI)	*p*‐value	HR (95% CI)	*p*‐value
Age (≤65/>65)	1.023 (1.010−1.036)	<0.001	1.024 (1.011−1.037)	<0.001
TNM stage (I/II/III/IV)	1.643 (1.028−1.752)	0.039	1.198 (0.885−1.622)	0.177
Histologic grade (G_1/2_/G_3/4_)	1.213 (0.816−1.801)	0.340	1.308 (0.871−1.963)	0.196
Risk score (H/L)	2.334 (1.817−2.997)	<0.001	2.361 (1.830−3.047)	<0.001

Abbreviations: GRGs, glycolysis‐related genes; H, high; L, low.

### 3.5 Validation of the nine‐GRG signature in predicting survival using Kaplan‐Meier curves

The clinical features of age, histological grade, and tumor stage represent predictive prognostic factors of OV after the performance of univariate Cox regression analysis of OS. Kaplan‐Meier curves revealed that clinical features that showed consistent results, namely patient age >65 years and disease stages III and IV, were associated with poor prognosis (Figure 5D,E).

**FIGURE 6:**
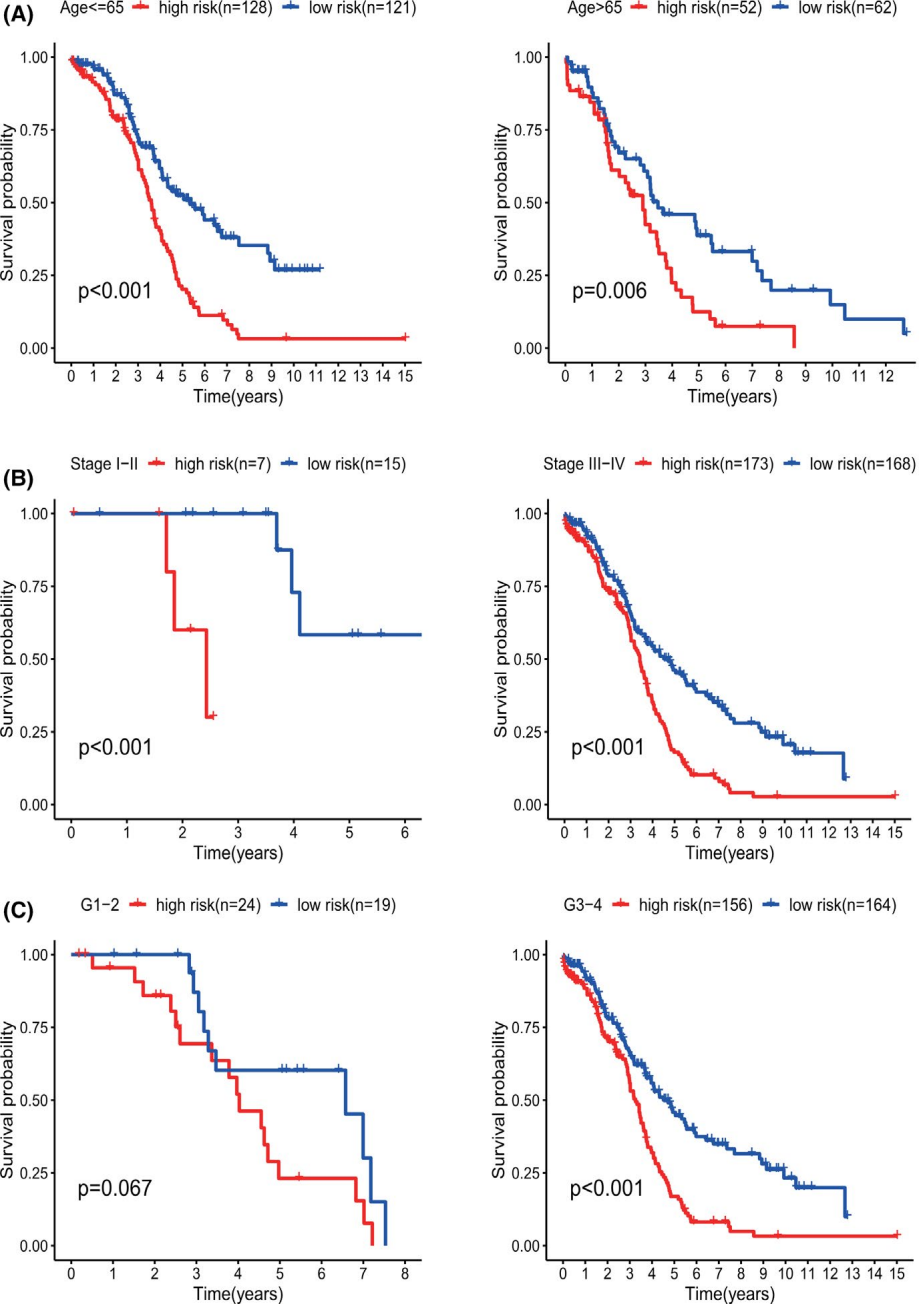
KM survival subgroup analysis of all patients with OV according to the GRG‐related gene signature stratified by clinical characteristics. (A) Age ≤ 65 years, age > 65 years. (B) Early stage (stage I–II), late stage (stage III–IV). (C) Low grade 1–2, High grade 3–4. GRGs, glycolysis‐related genes; OV, ovarian cancer

To test whether our nine‐GRG signature can play a role in different TNM stages, histological grades, and ages, a subgroup analysis was performed for each clinical feature. Kaplan‐Meier survival analysis demonstrated that the risk signature had a stable prognostic power and was applicable to patients with OV when they were stratified into different age and TNM stage groups (Figure 6A,6B). However, when the patients were stratified into high‐grade (grades 3 and 4) and low‐grade (grades 1 and 2) subgroups, the risk score of the nine‐gene signature remained an independent prognostic indicator in the high‐grade subgroup (*p* < 0.001) but not in the low‐grade subgroup (*p* = 0.067; Figure 6C). The risk model showed a more effective prediction in patients with high‐grade OV.

### 3.6 Comparison with other prognostic signatures

We compared our gene signature with other known prognostic signatures to assess the robustness of our model. To exclude the effect of heterogeneity, only signatures that were developed based on the TCGA database were included. The studies on markers for predicting specific types of prognosis for patients with OV were excluded from our comprehensive evaluation.^32^, ^33^, ^34^ Finally, 22 OS‐related prognostic signatures were included for comparison with our gene signature. The results demonstrated that our signature yielded remarkably good performance in predicting OS in patients with OV (Table 4). In our study, the AUCs of the signatures at 3, and 5 years were 0.709, 0.762, respectively, which were significantly higher than those of most hallmark predictive models. Table 4 shows that the AUCs of the other three prognostic signatures, namely, the 21 immune‐related gene signature,^35^ 17 immune‐related gene signature (0.754 at 3 years, 0.824 at 5 years)^36^ and 17 transcription factor ‐related gene signature (0.803 at 5 years),^37^ were comparable to the predictive capabilities of our predictive model and distinctly higher than those of other signatures, such as the epithelial‐mesenchymal transition related gene signature,^38^ TME‐related gene signature,^39^, ^40^ RNA‐binding protein‐related gene signature,^41^ energy metabolism‐related gene signature,^42^ autophagy‐related gene signature,^43^, ^44^ ferroptosis‐related gene signature,^45^ protein‐coding gene signature,^46^ and DNA methylated gene signatures.^47^ The larger the AUC value of a biomarker, the better was the predictive ability of the signature, indicating that our gene signature outperformed most of the other signatures in predicting OV prognosis.

**TABLE 4:** The area under the ROC curve (AUC) show the sensitivity and specificity of the known gene signatures in predicting the prognosis of OV patients

Author	Year	Gene signature	AUC for OS
Our study	2021	9 GRG signature	0.709 (3‐year), 0.762 (5‐year)
Cao T, et al.	2021	21 immune‐related gene signature	0.746 (1‐year), 0.735 (3‐year), 0.749 (5‐year)
He C, et al.	2021	6 RBP‐related gene signature	0.657 (3‐year), 0.718 (5‐year)
Li H, et al.	2021	17 TF‐related gene signature	0.803 (5‐year)
Yang L, et al.	2021	9 ferroptosis‐related gene signature	0.654 (1‐year), 0.664 (3‐year), 0.690 (5‐year)
An Y, et al.	2020	15 immune‐related gene signature	0.683 (5‐year)
Ding Q, et al.	2020	9 TMB‐related gene signature	0.684 (3‐year), 0.707 (5‐year)
Fan L, et al.	2020	18 m6A–related signature	0.58 (5‐year)
Guo Y, et al.	2020	3 TMB‐related gene signature	0.701 (3‐year), 0.727 (5‐year)
Lin H, et al.	2020	2 immune‐related gene signature	0.678 (3‐year), 0.620 (5‐year)
Meng C, et al.	2020	17 autophagy‐related lncRNA signature	0.731 (5‐year)
Pan X, et al.	2020	6 EMT gene signature	0.711 (5‐year)
Yan S, et al.	2020	5 immune infiltration‐related gene signature	0.704 (5‐year)
Zhang B, et al.	2020	17 immune‐related gene signature	0.755 (1‐year), 0.754 (3‐year), 0.824 (5‐year)
Zhang Q, et al.	2020	8 MRG signature	0.653 (1‐year), 0.68 (3‐year), 0.616 (5‐year)
Zheng M, et al.	2020	11 lipid metabolism gene signature	0.706 (2‐year), 0.694 (3‐year), 0.724 (5‐year)
Sun H, et al.	2019	14 DNA repair gene signature	0.759 (5‐year)
An Y, et al.	2018	8 autophagy‐related gene signature	0.703 (5‐year)
Guo Q, et al.	2018	5 TF‐related lncRNA signature	0.700 (5‐year)
Guo W, et al.	2018	5 DNA methylated gene signature	0.715 (5‐year)
Zhang J, et al.	2018	2 protein‑coding gene signature	0.642 (5‐year)
Liu L, et al.	2016	5 gene signature	0.670 (5‐year)
Zhou M, et al.	2016	8 lncRNA signature	0.705 (5‐year)

Abbreviations: EMT, epithelial–mesenchymal transition; MRG, metabolism‐related gene; OS, overall survival; RBP, RNA‐binding protein; TF, transcription factor; TME, tumor microenvironment.

## 4 DISCUSSION

Increasing attention has been paid to the global burden of OV. Despite current advances in surgery and chemotherapy, poor prognosis remains a major challenge.^3^ Because of heterogeneity and the lack of convenient and accurate biomarkers, the current prognostic tools for patients with OV have limited clinical predictive abilities.^2^, ^48^ Subtype identification, risk stratification, and characterization of the underlying mechanism are critical for the improvement of existing treatment methods, development of more precise and personalized therapies, and prolongation of survival time. Thus, a predictive model with a broad scope of application is needed to accurately predict OS in patients with OV and guide clinicians in providing targeted treatment and better prognosis. With the popular application of large databases, an increasing number of prognostic markers have been recognized.^8^, ^9^, ^26^ In recent decades, the metabolic processes in tumor microenvironment have gradually become research hotspots in tumor research and treatment.^49^ The Warburg effect is a hallmark of cancer research and plays an important role in promoting the occurrence and development of tumors. It has been observed that most tumor cells continue to rely on aerobic glycolysis for energy, even with adequate oxygen and nutrition.^50^ Thus, aerobic glycolysis promotes the rapid proliferation of cancer cells, progression of cancer, and resistance to apoptotic cell death.^51^ Previous studies have investigated the role of GRGs and glycolysis in the development of several cancers, and GRG models have been built successfully^14^, ^15^, ^16^; however, no related research on OV has been reported to date. Considering the poor survival and high mortality of patients with OV and the lack of comprehensive investigations on OV, we established a GRG‐based risk signature to predict the OS of patients with OV.

In this study, our predictive model consisted of a training set and three validation cohorts, which included 813 patients with OV. Nine genes with prognostic value for patients with OV were identified using univariate, multivariate, and LASSO Cox regression analyses. The results indicate that the nine‐GRG signature developed in this study significantly correlated with poor prognosis in OV. In addition, this risk signature remained an independent prognostic factor in multivariate Cox analyses. The results of survival analysis suggested that patients with high‐risk scores tended to have worse clinical outcomes. The nine‐gene model showed a better predictive ability than any single gene or clinicopathological factor. The model established in the present study is well suited for OS prediction. Nomograms have been constructed to predict various clinical endpoints in patients with different types of cancers.^52^, ^53^ Theoretically, a nomogram should be specific to each patient and thus be able to accurately predict specific clinical endpoints. In our study, a novel nomogram was constructed by combining a prediction model with clinical characteristics. The nomogram used complementary values of clinical characteristics and prediction model and provided better estimates of individual outcomes.

Gene signature could further assess the survival risk in patients with different clinical features (age, TNM stage, and histological grade). The risk model had effective prediction power for patients with diverse clinical characteristics, but its predictive power was limited in patients with a low histological grade, which should be explored in depth in the future. This result implies that the clinical application of genetic models is far‐reaching and the methods for predicting prognosis of patients in clinical settings will become more diverse, thus guiding clinicians to provide accurate and effective treatment.

To further explore the predictive ability of our signature, a comparison was performed among several significant molecular signatures that were employed for predicting OS in patients with OV. The included studies ^11^, ^35^, ^36^, ^37^, ^38^, ^39^, ^40^, ^41^, ^42^, ^43^, ^44^, ^45^, ^46^, ^47^, ^54^, ^55^, ^56^, ^57^, ^58^, ^59^, ^60^, ^61^ used models built based on the TCGA cohort and involved all types of breast cancer. The final results showed that our signature and three other prognostic signatures, namely, a 21 immune‐related gene signature,^35^ 17 immune‐related gene signature^36^ and 17 TF‐related gene signature^37^ performed better than the other hallmark signatures in the prediction of OS in patients with OV.^11^, ^35^, ^38^, ^39^, ^40^, ^41^, ^42^, ^43^, ^44^, ^45^, ^46^, ^47^, ^54^, ^55^, ^56^, ^57^, ^58^, ^59^, ^60^, ^61^ Additionally, Yu et al. has constructed a five GRG signature (ANGPTL4, PYGB, ISG20, SEH1L and IRS2) for patients with OV.^62^ The AUCs of the signature in Yu's study at 5‐years were 0.680. By contrast, besides the difference of database sources and grouping methods, in the process of screening our nine hub genes, we especially applied LASSO analysis, which was proved to be a scientific and effective screening method and it was widely used in many studies.^26^, ^31^, ^63^ Moreover, the AUC value shows that our signature (0.762 at 5 years) is better than Yu's model in predicting the 5‐year prognosis of OV patients. In addition to a larger sample size, our subgroup analysis showed that the nine‐GRG signature can perform better in high‐grade OV groups. Furthermore, we innovatively compared with other hallmark gene prediction models for OV. In addition to the four genes (ANGPTL4, PYGB, ISG20, and IRS2) we discovered together, we also discovered that another five genes (CITED2, LHX9, PC, TGFBI, and DDIT) are related to the prognosis of OV, which undoubtedly provides a favorable basis for future research. Therefore, our signature may help in enriching clinical prediction methods and developing more effective targeted therapies that contribute to improved prognosis.

Among the nine biomarker genes identified in the present study, DNA damage‐inducible transcript 4 (*DDIT4*), with high expression levels, actively responded to hypoxia‐inducible factor 1 and acted synergistically to regulate the generation of cell reactive oxygen species.^64^ As an oncogene,^64^, ^65^ the overexpression of *DDIT4* correlates with tumor progression and worse outcomes in several human cancers, including OV.^18^, ^66^, ^67^, ^68^ Brain‐type glycogen phosphorylase (*PYGB*) could regulate multiple biological characteristics of cancer cells, such as proliferation, invasion, and apoptosis, and metastatic phenotypes of several cancers.^69^, ^70^, ^71^, ^72^, ^73^, ^74^ *PYGB* regulates the Wnt/β‐catenin signaling pathway to achieve cancer‐promoting effects in OV,^75^ non‐small cell lung cancer,^76^ and gastric cancer.^77^ Insulin receptor substrate 2 (*IRS2*) mediates mitogenic and antiapoptotic signaling of insulin‐like growth factor 1 receptor, insulin receptor, and other oncoproteins^78^, ^79^ and is essential for cancer cell motility and metastasis.^80^, ^81^, ^82^ *IRS2* acts as an oncogene in OV and is involved in cell proliferation and ascites migration during OV progression.^83^, ^84^ Angiopoietin‐like 4 (*ANGPTL4*) has been reported to be involved in ferroptotic cell death and chemoresistance of epithelial OV.^85^ Moreover, large amounts have been detected in the malignant ascites of patients with serous OV.^86^ High *ANGPTL4* levels predict short relapse‐free survival in serous OV.^86^, ^87^ Studies have found that high promoter hypermethylation of transforming growth factor‐beta‐inducible gene (*TGFBI*) is involved in chemotherapy resistance of paclitaxel in OV.^88^, ^89^ A study showed that *TGFBI* and periostin predict poor prognosis in serous epithelial OV.^90^ Pyruvate carboxylase (*PC*) is a biotin‐containing enzyme that converts pyruvate to oxaloacetate and has been implicated in cancer progression. *PC* is strongly involved in tumorigenesis in several cancers, such as breast cancer, non‐small cell lung cancer, glioblastoma, renal carcinoma, and gallbladder cancer.^91^, ^92^, ^93^, ^94^ Moreover, *PC* may mediate the regulation of tankyrase (TNKS) in aerobic glycolysis and may be involved in the TNKS‐regulated development of OV, as its oncogenic activity is induced by TNKS activating Wnt/β‐catenin/snail signaling.^95^ Not much evidence has been accumulated on the following genes from basic research on OV. Interferon‐stimulated gene 20 (*ISG20*) is an RNA exonuclease^96^ that stimulates tumor progression in hepatocellular carcinoma, clear cell renal cell carcinoma, and glioma.^97^, ^98^, ^99^ The high expression level of ISG20 is associated with poor clinical outcomes in patients with OV.^99^ Cbp/p300‐interacting transactivator 2 (CITED2), a pleiotropic protein, has been reported to participate in several biological functions of cells, including transcription and differentiation. High CITED2 expression levels are correlated with poor patient survival in breast^100^ and prostate^101^ cancers. CITED2 participates in the regulation of the cell cycle, promotes cell proliferation, and plays an active role in the progression of lung cancer^102^, ^103^ and supports gastric cancer cell colony formation and proliferation.^104^ In addition, it is involved in resistance to platinum‐based chemotherapy in OV.^105^ LIM homeobox 9 (LHX9) is a developmentally expressed transcription factor^106^ that is strongly expressed in the ovarian surface epithelium.^107^ Previous research has shown that childhood malignant gliomas involve abnormal methylation and silencing of LHX9,^108^ and the relationship between ISG20, CITED2, and LHX9 with OV and its molecular mechanism must be examined in depth in future studies. We integrated the nine GRGs into a panel and established a novel multigene signature to predict the prognosis of OV. This signature showed a strong predictive ability and acted as an independent prognostic molecular factor in patients with OV.

Our study identify a GRG risk predictive signature using data from public database. The nine‐GRG risk model showed promising survival prediction ability for the prognosis of OV. Despite these promising results, there are certain limitations to our study. First, this was not a prospective study, and all patients with OV were identified from public databases. Second, the missing rate for the clinical characteristics was high, which decreased the statistical power in multivariable Cox regression analysis and the integrated prognostic model. Third, large‐scale multicenter cohorts are necessary to verify our findings and further basic experiments in our hospital are needed to explore the functional roles of the GRGs involved in the initiation and development of OV. In addition, the gene signature performed more effectively in patients with high‐grade OV than in patients with low‐grade OV, and the reason for this should be investigated in detail in the future. Finally, our model cannot predict recurrence and distant metastasis in patients with OV owing to the lack of relevant data in the TCGA database. To further validate the utility of this risk model, we have undertaken the collection of clinical data and specimens.

## 5 CONCLUSION

We constructed a valid, innovative, and reliable nine‐GRG prognostic model (*ISG20*, *CITED2*, *PYGB*, *IRS2*, *ANGPTL4*, *TGFBI*, *LHX9*, *PC*, and *DDIT4*) to predict patient outcomes in OV. Moreover, our signature is an independent and important risk factor for OV. The predictive capability of this model in OV requires further testing to improve prognostic stratification and treatment management.

## ETHICS APPROVAL AND CONSENT TO PARTICIPATE

This study has been proved by the Institutional Review Board of the First Affiliated Hospital of Zhejiang University in Zhejiang Province (Hangzhou, China).

## CONSENT FOR PUBLICATION

Written informed consent for publication was obtained from all participants.

## CONFLICT OF INTEREST

The authors declare that they have no conflicts of interest.

## Supporting information

Table S1

Table S2

## Data Availability

The datasets generated and analyzed during the current study are available in the TCGA (http://cancergenome.nih.gov/abouttcga), GEO (https://www.ncbi.nlm.nih.gov/geo/) and cBioPortal (http://www.cbioportal.org) databases. ZJD and HFK had full access to all the data in the study and takes responsibility for the integrity of the data and the accuracy of the data analysis.
