# Supplementary material for: Identification of a glycolysis‐related gene signature for survival prediction of ovarian cancer patients
Source: Cancer Med. 2021 Oct 5;10(22):8222–37. doi: 10.1002/cam4.4317 (PMC8607265; doi:10.1002/cam4.4317)
Supplement: Supplementary file 1 — Table S1 [file CAM4-10-8222-s001.docx]

**Table S1.The list of the glycolysis-related genes.**

| **Gene Names** |
| --- |
| ABCB6  ADORA2B  AGL  AGRN  AK3  AK4  ALG1  ANG  ANGPTL4  ANKZF1  ARPP19  ARTN  AURKA  B3GALT6  B3GAT1  B3GAT3  B3GNT3  B4GALT1  B4GALT2  B4GALT4  B4GALT7  BIK  BPNT1  CACNA1H  CAPN5  CASP6  CD44  CDK1  CENPA  CHPF  CHPF2  CHST1  CHST12  CHST2  CHST4  CHST6  CITED2 |
| **Gene Names** |
| CLDN3  CLDN9  CLN6  COG2  COL5A1  COPB2  CTH  CXCR4  CYB5A  DCN  DDIT4  DEPDC1  DLD  DPYSL4  DSC2  ECD  EFNA3  EGFR  EGLN3  ELF3  ENO1  ENO2  ERO1A  EXT1  EXT2  FAM162A  FKBP4  FUT8  G6PD  GAL3ST1  GALE  GALK1  GALK2  GAPDHS  GCLC  GFPT1  GLCE  GLRX |
| **Gene Names** |
| GMPPA  GMPPB  GNE  GNPDA1  GOT1  GOT2  GPC1  GPC3  GPC4  GPR87  GUSB  GYS1  GYS2  HAX1  HDLBP  HMMR  HOMER1  HS2ST1  HS6ST2  HSPA5  IDH1  IDUA  IER3  IGFBP3  IL13RA1  IRS2  ISG20  KDELR3  KIF20A  KIF2A  LCT  LHPP  LHX9  MDH1  MDH2  ME1  ME2  MED24 |
| **Gene Names** |
| MERTK  MET  MIF  MIOX  MPI  MXI1  NANP  NASP  NDST3  NDUFV3  NOL3  NSDHL  NT5E  P4HA1  P4HA2  PAM  PAXIP1  PC  PDK3  PFKFB1  PFKP  PGAM1  PGAM2  PGK1  PGLS  PGM2  PHKA2  PKM  PKP2  PLOD1  PLOD2  PMM2  POLR3K  PPFIA4  PPIA  PPP2CB  PRPS1  PSMC4 |
| **Gene Names** |
| PYGB  PYGL  QSOX1  RARS1  RBCK1  RPE  RRAGD  SAP30  SDC1  SDC2  SDC3  SDHC  SLC16A3  SLC25A10  SLC25A13  SLC35A3  SLC37A4  SOD1  SOX9  SPAG4  SRD5A3  STC1  STC2  STMN1  TALDO1  TFF3  TGFA  TGFBI  TKTL1  TPBG  TPI1  TPST1  TSTA3  TXN  UGP2  VCAN  VEGFA  VLDLR |
| **Gene Names** |
| XYLT2  ZNF292  AAAS  ADPGK  ALDOA  ALDOB  ALDOC  BPGM  ENO1  ENO2  ENO3  GAPDH  GAPDHS  GCK  GCKR  GNPDA1  GNPDA2  GPI  HK1  HK2  HK3  NDC1  NUP107  NUP133  NUP153  NUP155  NUP160  NUP188  NUP205  NUP210  NUP214  NUP35  NUP37  NUP43  NUP50  NUP54  NUP58  NUP62 |
| **Gene Names** |
| NUP85  NUP88  NUP93  NUP98  NUPL2  PFKFB1  PFKFB2  PFKFB3  PFKFB4  PFKL  PFKM  PFKP  PGAM1  PGAM2  PGK1  PGK2  PGM2L1  PGP  PKLR  PKM  POM121  POM121C  PPP2CA  PPP2CB  PPP2R1A  PPP2R1B  PPP2R5D  PRKACA  PRKACB  PRKACG  RAE1  RANBP2  SEC13  SEH1L  TPI1  TPR  ACSS1  ACSS2 |
| **Gene Names** |
| ADH1A  ADH1B  ADH1C  ADH4  ADH5  ADH6  ADH7  AKR1A1  ALDH1A3  ALDH1B1  ALDH2  ALDH3A1  ALDH3A2  ALDH3B1  ALDH3B2  ALDH7A1  ALDH9A1  ALDOA  ALDOB  ALDOC  BPGM  DLAT  ENO3  FBP1  FBP2  G6PC  G6PC2  GALM  GAPDH  GCK  GPI  HK1  HK2  HK3  LDHA  LDHAL6A  LDHAL6B  LDHB |
| **Gene Names** |
| LDHC  PCK1  PCK2  PDHA1  PDHA2  PDHB  PFKL  PFKM  PGAM4  PGK2  PGM1  AC016586.1  ACTN3  ADPGK  ARNT  MLXIPL  MYOG  NCOR1  NDC1  NUP107  NUP133  NUP153  NUP155  NUP160  NUP188  NUP205  NUP210  NUP214  NUP35  NUP37  NUP43  NUP50  NUP54  NUP58  NUP62  NUP85  NUP88  NUP93 |
| **Gene Names** |
| NUP98  NUPL2  OGDH  OGDHL  OGT  P2RX7  PFKFB2  PFKFB3  PFKFB4  POM121  POM121C  PPARA  PPARGC1A  PRKAA1  PRKAA2  PRKAG1  PRKAG2  PRKAG3  PRXL2C  RAE1  RANBP2  SEC13  SEH1L  STAT3  TIGAR  TREX1  ZBTB20  ZBTB7A  PGM2L1  HKDC1  HTR2A  IGF1  INS  INSR  JMJD8  ENO4  ENTPD5  ESRRB |
| **Gene Names** |
| GCKR  GPD1  HDAC4  HIF1A  CBFA2T3  DHTKD1  EIF6 |
