# Supplementary material for: Identification of a glycolysis‐related gene signature for survival prediction of ovarian cancer patients
Source: Cancer Med. 2021 Oct 5;10(22):8222–37. doi: 10.1002/cam4.4317 (PMC8607265; doi:10.1002/cam4.4317)
Supplement: Supplementary file 2 — Table S2 [file CAM4-10-8222-s002.docx]

Table S2. Clinical characteristic descriptions for ovarian cancer patients in validation datasets.

| **Characteristics** | | **GSE26193** | **GSE30161** | **GSE63885** |
| --- | --- | --- | --- | --- |
| Number of samples | | 107 | 58 | 101 |
| Median OS (month) | | 42 (95% Cl, 9.6-108） | 50 (95% CI, 32–68) | 72 (95% CI, 12-62) |
| Median PFS (month) |  | 19 (95% Cl, 3.6-108） | 13 (95% CI, 10–16) | - |
| Number of Death | | 76 (71%) | 36 (62%) | 66 (65%) |
| Age (years) |  | - | 62 (38–65) | - |
| Histology type | |  |  |  |
| Serous |  | 79 (74%) | 47 (81%) | 74 (73%) |
| Endometroid | | 8 (7%) | 1 (2%) | 12 (12%) |
| Clear cell |  | 6 (6%) | 5 (9%) | 9 (9%) |
| Mucinous |  | 8 (7%) | 2 (3%) | 0 |
| Other |  | 4 (4%) | 3 (5%) | 6 (6%) |
| FIGO stage | |  |  |  |
| Ⅰ |  | 21 (20%) | - | 29 (29%) |
| II |  | 10 (9%) | - | 3 (3%) |
| III |  | 59 (55%) | - | 59 (58%) |
| IV |  | 17 (16%) | - | 10 (10%) |
| Grade |  |  |  |  |
| 1 |  | 7 (7%) | 1 (2%) | 24 (23%) |
| 2 |  | 33 (31%) | 2 (3%) | 10 (10%) |
| 3 |  | 0 | 50 (86%) | 48 (48%) |
| 4 |  | 67 (63%) | 5 (9%) | 19 (19%) |
| CHT-response | |  |  |  |
| CR |  | - | 32 (55%) | 58 (57%) |
| PR, PD |  | - | 23 (40%) | 36 (36%) |
| SD |  | - | 3 (5%) | 7 (7%) |

Abbreviation: PFS:  Progression Free Survival; OS: Overall Survival; CI: Confidence Interval; CR: Complete Response; PR: Partial Response; PD = Progressive Disease; SD, stable disease.
